# Diagnosis and Treatment of a Morel-Lavallée Lesion in the Lateral Knee With Point-of-Care Ultrasonography

**DOI:** 10.7759/cureus.39118

**Published:** 2023-05-17

**Authors:** Kathryn B Vess, Jeff Cashman, Jacob Ringenberg, Jordan Freeland

**Affiliations:** 1 Sports Medicine, Edward Via College of Osteopathic Medicine, Spartanburg, USA; 2 Family Medicine, Edward Via College of Osteopathic Medicine, Spartanburg, USA; 3 Sports Medicine, Self Regional Healthcare, Greenwood, USA; 4 Family Medicine, Self Regional Healthcare, Greenwood, USA

**Keywords:** primary percutaneous intervention, trauma pediatric, morel-lavallee lesion, knee trauma, point-of-care ultrasonography

## Abstract

A 14-year-old male presented to the sports medicine clinic for evaluation of right lateral knee pain for three weeks after he took a forceful blow to his right lateral knee during a football game. He reported swelling and bruising and increasing pain since then. Physical exam was significant for an area of fluctuance that was approximately 20 cm in length and 10 cm in width overlying the lateral right knee with associated ecchymosis and decreased sensation. The remainder of the exam was benign. Point-of-care ultrasound showed a large hypoechoic space overlying the lateral knee consistent with a Morel-Lavallée lesion (MLL). Twenty-six milliliters of serosanguinous fluid were aspirated from between the fascial planes, deep to subcutaneous fat but superficial to quadriceps muscles, under ultrasound guidance. The lesion was sclerosed with 1 cc 1% lidocaine without epinephrine and 4 cc dexamethasone 4 mg/mL, and the patient was given compression wrappings to wear for the next four weeks. MLLs are collections of fluid that occur between different planes of subcutaneous tissue following blunt force or shearing trauma. The general mechanism of injury is a closed degloving injury that occurs following damage to the potential space between layers of fascia, dermis, and subcutaneous fat. MLLs are relatively rare lesions and, when identified, are most frequently found in the proximal thigh and associated with serious underlying bony fractures. MLLs are uncommon and difficult to diagnose due to their nonspecific findings of fluctuance, pain, and bruising. This case is unique in its presentation of an isolated MLL in the lateral knee. Early diagnosis and intervention of these lesions prevent further sequelae.

## Introduction

Morel-Lavallée lesions (MLLs) were first described in 1853 by French physician Maurice Morel-Lavallée [1]. These lesions are described as collections of fluid that occur between different planes of subcutaneous tissue following blunt force or shearing trauma [2]. The general mechanism of injury is a closed degloving injury that occurs as a result of damage to the potential space between layers of fascia, dermis, and subcutaneous fat [1-2]. The initial damage can lead to the shearing of small vessels contained therein, which causes a collection of blood between the fascial planes [3]. This initial injury can lead to edema, pain, and ecchymosis [3]. As the injury progresses, the blood is reabsorbed into the tissues leaving a serosanguinous fluid in its place [3]. If the lesion persists, a hemosiderin layer will separate from the liquid and can form a capsule around the fluid collection [3].

These lesions are difficult to diagnose; therefore, it is difficult to estimate their annual occurrence. Often these lesions are identified incidentally during operations to address orthopedic injuries due to underlying fractures, most commonly of the greater trochanter or femoral neck [4]. MLLs predominantly follow shearing trauma overlying the greater trochanter [4]. However, these lesions have also been identified in the knees, buttocks, lumbosacral area, lower leg, and head [5-6]. Approximately 15.7% of identified MLLs occur in the knee, with a greater predilection for the medial than lateral side due to increased resistance to shearing forces before separation occurs [5-7].

The most common clinical presentation of MLLs are bruising, fluctuance of the skin, skin mobility, and decreased sensation overlying the area of fluctuance [8]. Many presenting lesions have coexisting ligamentous or bony injuries as a result of the initial trauma that also requires assessment and diagnosis [4]. After underlying ligamentous or bony abnormalities have been ruled out with a thorough physical exam and imaging studies, the modalities of choice to diagnose MLL is either a point-of-care ultrasound or an MRI depending on the resources available and the associated concern for structural abnormality [9].

The treatment of an MLL is dependent on the size of the lesion and the presence or absence of a capsule surrounding the lesion [8]. Lesions smaller than 400 mL can be drained percutaneously under ultrasound guidance [8,10]. After sufficient drainage of the lesion, it can then be infiltrated with a sclerosing agent such as doxycycline or dextrose to prevent continued leakage of fluid into the lesion and to encourage the detached fascial layers to reapproximate [11]. Sclerosing agents are caustic materials that work by inducing local inflammation in the subcutaneous layers that lead to deposition of collagen and fibrin. This encourages the layers to coalesce without pockets of fluid being allowed to re-accumulate [11]. Other agents for sclerosis include ethanol, bleomycin, and OK-432 [11]. Of these, doxycycline and dextrose represent the most cost-efficient options with the most favorable side effect profiles, making them the preferred agents in orthopedic sclerosis [12]. Following aspiration of the lesion, compression dressing is applied daily to help mechanically align the layers of fascia [10]. Compression alone is a reasonable treatment, particularly in patients unable to tolerate the aspiration process; however, the healing time is increased, and they have a significant risk of re-accumulation of fluid [10]. Lesions larger than 400 mL typically do not respond well to percutaneous intervention and are instead referred for surgical repair [8]. Open drainage or mass resection can be augmented with open surgical drains, fibrin glue, or quilting sutures to help prevent poor surgical outcomes and lesion recurrence [8].

The most common complication of MLLs is recurrence with up to 56% of lesions reoccurring in the first six months following percutaneous drainage or compression-only treatment [8]. Common causes of recurrence in the nonoperative patient include large lesions and the presence of a capsule surrounding the lesion [8]. Other complications associated with MLL are skin necrosis, infection, and the formation of a pseudocyst after aspiration [3,8]. Most patients require close follow-up in the weeks following intervention to assess for recurrence, infection, and signs of skin breakdown [10]. However, with early intervention and treatment, patients can recover well with few adverse events [10].

## Case presentation

A 14-year-old male with no significant past medical history presented to the clinic for evaluation of right lateral knee pain for three weeks. The patient is the starting tight end for his high school football team and reported that his pain began during a football game three weeks ago when he took a direct blow to his lateral knee from an opposing player. He reported he was able to walk after the initial hit without any feelings of joint instability, but over the next three weeks began to have increasing pain, swelling, and bruising of the right lateral knee. He reported the pain was worse when he was hit on that side at subsequent football practices and games and was relieved with Tylenol 325 mg every four to six hours. 

In the clinic, the patient was ambulating with an antalgic gait and had a large area of fluctuance on the right lateral knee that was approximately eight inches in diameter and extended from his distal thigh to midway down his calf. The area of fluctuance was tender to palpation and had diffuse ecchymosis. Additionally, the patient reported decreased sensation overlying the lesion. Deep tendon reflexes and sensation over the remainder of his lower extremity were intact. Additional exams were done to assess for ligamentous instability due to the nature of the injury; however, the patient did not have any evidence of anterior cruciate ligament (ACL), posterior cruciate ligament (PCL), medial collateral ligament (MCL), lateral collateral ligament (LCL), or meniscus injury.

**Figure 1:**
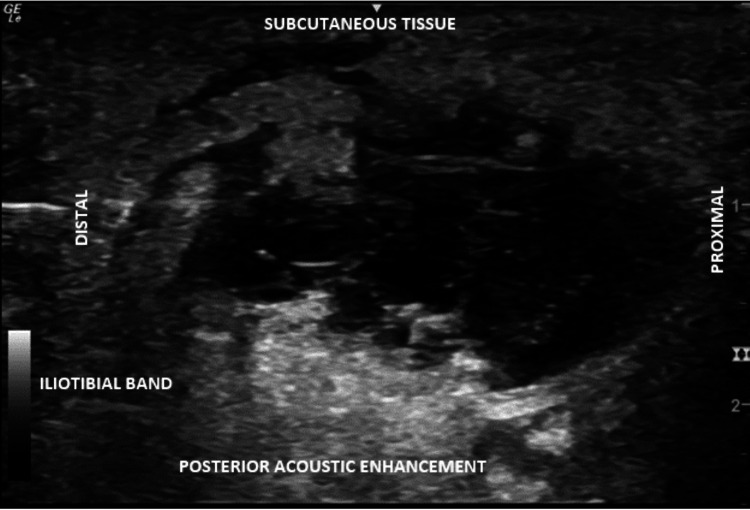
Knee US: US imaging of the right lateral knee with a large hypoechoic space between subcutaneous layers of tissue being visualized using the short axis.

**Figure 2:**
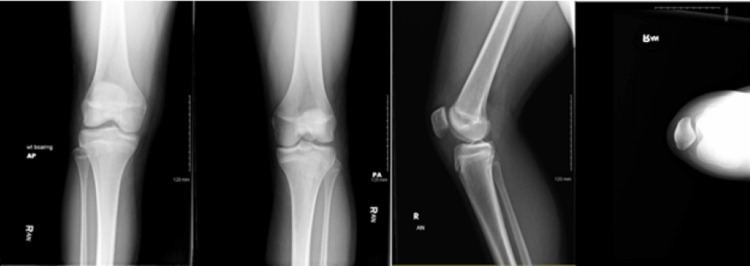
Knee XR: (a) AP right knee, (b) PA right knee, (c) lateral view right knee, and (d) sunrise view right knee. AP, anteroposterior; PA, posteroanterior

The knee X-ray was negative for acute bony abnormality (Figure 1). A point-of-care ultrasound in the office showed a large hypoechoic mass between distinct fascial planes on the lateral aspect of the right knee (Figure 2). This presentation was consistent with an MLL. 

The lesion was aspirated percutaneously under ultrasound guidance. Approximately 26 mL of serosanguinous fluid was drained from the patient’s MLL overlaying the knee. The lesion was then injected with a combination of 5 mL of 50% dextrose and 5 mL lidocaine 1%. This fluid was massaged into the lesion for one minute, and then the fluid was re-aspirated. A compressive wrapping was placed with instructions to maintain compression over the area and to avoid contact sports for one week. The aspirated fluid was cultured and showed no growth at five days.

The patient was reassessed one week later and had no evidence of fluctuance. A follow-up point-of-care ultrasound was negative for fluid re-accumulation.

## Discussion

The diagnosis of MLL is difficult to establish due to the lack of distinguishing characteristics of the lesions on the exam [3]. Many lesions are not discovered until operative treatment is being performed for other causes and they are discovered incidentally [9,13]. Patients who present after blunt force trauma, particularly when a shearing force is described, should have a physical exam to look for fluctuant skin, skin hypermobility, ecchymosis, and decreased sensation over the injury. These lesions are visible on MRI; however, if MRI is determined not to be necessary due to the lack of clinical concern for associated bony or ligamentous injury, then an ultrasound is diagnostic [9,13]. On ultrasound, the lesions can be identified by anechoic or hypoechoic spaces separated by fascial planes and other subcutaneous layers of tissue [14-15]. The fluid accumulations will not be located intra-articularly [14-15].

If identified, it is important to treat the lesion rapidly to prevent sequelae, including further fluid accumulation, skin necrosis and breakdown, and infection [8]. Lesions smaller than 400 mL of fluid by ultrasound measurements can be either treated with a compression dressing or percutaneously drained and sclerosed [9,10]. Dextrose is cost-effective, has a low likelihood of adverse events, and is readily available in most clinical settings [16]. Following treatment, continued compressive wrappings and prevention of re-injury are paramount to avoid the recurrence of the injury. Recurrence is a common complication that occurs in up to 56% of nonsurgically treated MLL [8,10].

## Conclusions

MLLs are difficult to diagnose. The use of ultrasound can identify MLL even in the absence of associated bony or ligamentous injuries. These lesions are benign when they are identified and treated promptly with a low risk of complications. The patient, in this case, healed well without any instances of fluid re-accumulation and was able to return to play in his school’s football playoffs.
